# Effect of Aging on Chemical Composition and Rheological Properties of Neat and Modified Bitumen

**DOI:** 10.3390/ma12244066

**Published:** 2019-12-05

**Authors:** Rita Kleizienė, Miglė Panasenkienė, Audrius Vaitkus

**Affiliations:** Road Research Institute, Vilnius Gediminas Technical University, Linkmenu str. 28, LT-08217 Vilnius, Lithuania; migle.panasenkiene@vgtu.lt (M.P.); audrius.vaitkus@vgtu.lt (A.V.)

**Keywords:** bitumen, asphalt binder, oxidative aging, aging index, colloidal stability, rheology

## Abstract

The aim of this research was to define the effect of oxidative aging on the chemical and rheological properties of neat and styrene-butadiene-styrene (SBS) polymer-modified bitumen. The experimental research had two objectives: firstly, the short and long-term effects of aging on the properties of neat and polymer-modified bitumen were investigated. Then, the aging indexes based on chemical and rheological properties to describe the age of unknown bitumen were established. Aging characteristics such as the Gaestel index, sulfoxide and carbonyl indexes, linear viscoelastic strain range, crossover temperature, and Glover–Rowe parameter were analysed for laboratory aged and naturally aged neat and polymer-modified bitumen. The functional composition of aged bitumen was evaluated by measuring absorption with Fourier transform infrared (FT-IR) attenuated total reflection (ATR) spectrometer. The saturates, aromatics, resins, and asphaltenes (SARA) fractions were determined with thin layer chromatography with flame-ionization detection (TLC-FID) to determine the colloidal instability index (Gaestel index). Finally, the complex shear modulus was determined with dynamic shear rheometer (DSR) to evaluate the influence of aging on the bitumen mechanical performance.

## 1. Introduction 

Almost 95% of the bitumen produced worldwide is used to construct roads with asphalt pavement [1]. The asphalt pavement industry leads the vehicles transportation sector because of comfortable driving, as well as easy and fast construction, maintenance, and repair activities. However, due to the nature of bitumen (asphalt binder), the asphalt pavement is subjected to more frequent repairs compared to concrete pavements [2]. Bitumen is an organic material, which in contact with atmospheric oxygen, heat, and solar radiation (ultraviolet light) hardens and ages, resulting in changes in the physical and rheological properties as well as the deterioration of asphalt pavement. Bitumen aging and rejuvenation phenomena are still under research focus, due to the increasing usage of reclaimed asphalt and asphalt pavement recycling [3,4,5]. With increasing concerns about environmental preservation, the usage of reclaimed asphalt pavement (RAP) for road construction and maintenance activities will increase from 20–30% to 80–100% [5,6,7]. In order to apply the high reclaimed asphalt content mixture, the rejuvenators or recycling agents have to be used to restore the aged binder properties. To select the effective type of rejuvenator and quantity, the bitumen aging phenomena have to be understood to assure the long-term performance of asphalt pavement. 

Bitumen consists of various sizes, aromaticity, and polarity hydrocarbon molecules, which due to complexity are hardly analysed individually; therefore, the chemical composition of bitumen is usually analysed through fractionation or functional groups and their ratio. The classical theory of bitumen composition is based on a colloidal system consisting of higher molecular weight asphaltenes dispersed in lower molecular weight maltenes (resins, aromatics, saturates) [8,9]. According to the colloidal composition, there are three types of bitumen behaviours [10]: sol (viscous), sol–gel (viscoelastic), and gel (elastic). Bitumen colloidal stability was determined by using the instability index I_c_—also called the Gaestel index [11]—which increases with an increased level of asphaltenes [12]. Thus, the asphaltenes are the most important components of a colloidal system of bitumen, whose quantity and interaction with the resins, aromatics, and saturates defines the rheological properties of bitumen. Corbett and Merz (1975) determined that the chemical composition changes in a similar pattern: the aromatics generate resins, which in turn generate asphaltenes [13]. The saturates remain essentially unchanged or slightly changed because of the low chemical reactivity [1,14,15]. However, the molecular structure of bitumen changes with the interaction of time, temperature, and oxygen; in other words, the quantity of fractional groups is diverse. In addition, the chemical changes during the aging process vary for different types of bitumen; they are subjected by production technology and different crude oil sources. The most significant properties linking the structure of the bitumen with its behaviour and rheology are the glass transition temperature of the maltenes and the effective content of asphaltenes [1]. These affects can be determined using scattering techniques (such as Atomic Force Microscopy (AFM), Scanning Electron Microscopy (SEM) and Fluorescence Microscopy, X-ray and other) and nuclear magnetic relaxometry, which are used for deeper analysis in ageing and rejuvenation of bitumen [16].

Oxidative hardening is imposed on the polar, oxygen-containing chemical components on bitumen molecules, causing increased molecular interactions and resulting in bitumen hardening [17,18,19]. Bitumen aging takes place during bitumen production (in refinery), storage, and transportation, asphalt mixing, asphalt mixture transportation and compaction, as well as from environmental impact over the pavement service life. Due to oxidative aging, the molecular interaction changes, affecting the bitumen and asphalt mixture behavior [17,20], which can be identified from the chemical functional groups such as carbonyl, sulfoxide, aromaticity, and hydroxyl. In addition, bitumen aging phases can be defined by fractional composition by the increasing molecular polarity of saturates, aromatics, resins, and asphaltenes [21]. With regard to chemical composition, a shift within the SARA (saturates, aromatics, resins and asphaltenes) fractions can be observed with an increasing content of asphaltenes and decreasing content of aromatics over time [22,23,24]. These factors cause an increase in the viscosity of the bitumen and consequential stiffening of the mixture. Other factors may also contribute to aging, such as molecular structuring over time (steric hardening) and actinic light (primarily ultraviolet radiation). Oxidation, volatile loss, and steric hardening tend to be universally accepted as the three dominant factors affecting age hardening [25,26]. 

To increase bitumen performance and durability, neat bitumen is modified by adding different types and quantities of polymers, elastomers, epoxide and/ or nanoclay additives [1,27,28]. Modified bitumen behaves and ages differently from neat bitumen [29], but the change of bitumen components may result in the degradation of polymers [30]. In terms of polymer-modified bitumen (PMB) aging, the structure also changes; since asphaltenes and polymers do not mix, a phase separation occurs, leaving on one side the polymer swollen by the aromatics components of the maltenes and on the other side the asphaltenes in the remaining maltenes [31]. As a consequence of the large quantity of aromatics required to swell the polymer, the matrix becomes depleted in maltenes and hence enriched in asphaltenes. Thus, the increased asphaltenes concentration generates a global hardening of the matrix, i.e., increases the high-temperature modulus of SBS-modified bitumen [32]. The different types of anti-aging additives were investigated to enable bitumen and polymers to resist the aging [33,34]. So, the need for new improved rejuvenation methods of modified bitumen (asphalt binder) is still relevant, especially by improving the adhesion between bitumen and aggregates as well as resistance to aging, assuring recyclability, and lowering modification costs.

There are several experimental tests to investigate bitumen resistance to aging by testing the chemical, physical, and rheological properties change after several aging levels. The changes in the bitumen were assessed by aging index measurements and chemical composition studies [35]. Mousavi et al. [36] determined that the ratio of polar components to nonpolar ones is higher in oxidized asphalt compared to virgin asphalt. Themeli et al. [37] analyzed the quantification of aging degree based on molecular weight distributions. Molecular weight distributions before and after aging were determined by the *δ*-method and by gel permeation chromatography (GPC) analyses. A new parameter, the aging molecular-distribution shift (AMDS), was proposed for the evaluation of molecular evolutions induced by aging. The results showed that molecular evolutions due to aging are directly responsible for the observed evolutions of the mechanical properties. Feng et al. [38] evaluated the correlation between colloidal chemistry and the aging properties of bitumen. The aging properties were compared using bitumen’s different physical parameters and colloidal indices [38]: penetration retention rate (PRR), ductility retention rate (DRR), viscosity aging index, and colloidal indices of neat and aged bitumen. The colloidal instability index is used to compare bitumen from different crude oil sources or analysing resistance to aging according to Equations (1) to (3) [38]:

(1) $$I_{p}=\frac{R_{e}}{A_{s}}*100\%,$$

(2) $$I_{s}=\frac{A_{s}}{\left(S_{a}+A_{r}+R_{e}\right)}*100\%,$$

(3) $$I_{c}=\frac{\left(A_{r}+R_{e}\right)}{\left(S_{a}+A_{s}\right)}*100\%,$$

where *Ip, Is,* and *Ic* are the colloidal indexes which show correlation with the penetration index, softening point and viscosity respectively; *S_a_*, *A_r_*, *R_e_*, and *A_s_* are the content of saturates, aromatics, resins, and asphaltenes, respectively (%).

The rheological properties of bitumen change due to age can be determined by analysing the penetration, softening point, viscosity, compliance, and shear modulus. Rheological properties describe the deformation or flow manner of bitumen in terms of time [39], and since bitumen performance is time and temperature dependent, it is attributed to the thermorheological simple materials group [40]. Bitumen is affected by time, heat, oxygen, and sunlight radiation changes, resulting in the hardening degrees of penetration, an increase in the softening point [41], and consequently an increase of the penetration index (PI) [42]. The concept of PI was developed for neat bitumen and not always fits for modified bitumen because of the large polymer structures (GEL type); therefore, the temperature susceptibility should be calculated based on the penetration determined at two temperatures. 

The master curve of complex shear modulus describes the viscoelastic behaviour of the bitumen as a function of both temperature and loading frequency, based on the time–temperature superposition principle [43,44,45,46]. After shifting measured $\left|G^{*}\right|$ data, the Richards generalized sigmoidal function was fitted for processing the qualitative parameters [46,47]. The mathematical representation of the Richards generalized sigmoidal function is defined [48]:

(4) $$log\left|G^{*}\right|=\delta +\frac{\alpha }{{\left(1+\lambda e^{\left(\beta +\gamma \left(log\omega_{r}\right)\right)}\right)}^{1/\lambda }}$$

where $\left|G^{*}\right|$ is the complex shear modulus, δ is the lower asymptote or the minimum value of $\left|G^{*}\right|$, δ+α is the upper asymptote or the maximum value of $\left|G^{*}\right|$, and λ, β, and γ are the shape parameters defining the function between the asymptotes and the location of the inflection point.

Based on the master curves of complex shear modulus, the Glover–Rowe (G-R) parameter and crossover temperature (T_δ = 45°_) can be estimated and used as representative indicators for evaluation of the rheological parameters. We evaluate the bitumen aging susceptibility to embrittlement and cracking at intermediate temperatures with G-R parameters [49,50], which were obtained at 15 °C and 0.005 rad/s according to Equation (5):

(5) $$G-R=\frac{G^{*}\times {\left(cos\left(\delta \right)\right)}^{2}}{sin\left(\delta \right)}.$$

According to Rowe [49,50], the warning and danger limit (zone) ranges for the G-R parameter are greater than 180 kPa and 600 kPa, which correspond to ductility values of 5 cm and 3 cm, respectively. It is accepted that G-R represents pavement resistance to ravelling and cracking influence by the oxidative aging of bitumen. 

Garcia Cucalon et al. [51] claimed that there is a correlation between G-R and T_δ = 45°_, and suggested to look for an initial threshold for T_δ = 45°_, which should be adjusted corresponding to the asphalt mixture properties and climate. Researchers [51] used the T_δ = 45°_ value together with the high-temperature performance grade and ΔT_c_ to determine the aging and rejuvenation ratio of the bitumen binder.

These bitumen aging indexes can be also used to determine the rejuvenator effect to recover the aged bitumen properties. However, this research aims to develop an understanding of the effect of oxidative aging on the chemical and rheological properties of neat and polymer-modified bitumen. The following objectives were established:

- Investigate the effect of short and long-term aging on the properties of neat and polymer-modified bitumen.
- Establish the aging indexes based on chemical and rheological properties to describe the aging processes of unknown bitumen.

To meet those objectives, the aging effects of laboratory aged and naturally aged neat and polymer-modified bitumen were analysed. The bitumen type (grade) for laboratory aging was selected considering the historical design data of road construction. The naturally aged bitumen was taken from several distressed pavements that required rehabilitation. 

## 2. Materials and Methods

### 2.1. Materials and Sample Preparation

Original (unaged) neat 70/100 and polymer-modified PMB 45/80-55 (SBS elastomer polymer) base bitumen were used to determine the aging effect on bitumen properties. The summary of investigated bitumen is presented in Table 1. 

#### 2.1.1. Laboratory-Aged Bitumen Procedure

The neat bitumen 70/100 and SBS polymer-modified bitumen PMB 45/80-55 were aged in these steps: (1) short-term aging to simulate the aging effects due to asphalt mixture production and layer compaction; (2) long-term aging to simulate 5–10 years of pavement in service; and (3) extended long-term aging to simulate 10–15 years of pavement in service. The rolling thin film oven test (RTFOT and RT) was used for the short-term aging of bitumen samples according to EN 12607-1 [52]. The bottles with 35 ± 0.5 g of bitumen were placed in an oven with a carousel at 163 °C, where hot air was periodically injected inside at a rate of 4000 ± 200 mL/min for 75 min. 

The pressure aging vessel (PAV) test was used for long-term and extended long-term bitumen aging after RTFOT according to EN 14769 [53]. The pans with 50 ± 0.5 g of bitumen were placed in a pressure chamber. The long-term aging (PAV I and P1) and extended long-term aging (PAV II and P2) were performed at 90 °C and 2.1 ± 0.1 MPa air pressure for 22 h and for 44 h, respectively.

#### 2.1.2. Field-Aged Bitumen Recovery Procedure 

The field-aged unknown type bitumen was recovered asphalt wearing layer of five different roads and streets pavements. Field cores were taken in 2017 after 12–19 years in service. The 150-mm diameter cores were sliced in 30-mm thick parts. Top slices of the asphalt surface layer were collected for bitumen extraction and recovery. The bitumen extraction involved heating top-sliced asphalt cores at 140 °C and preparing the loose mix. After cooling down, the loose mix was filled and kept with toluene reagent for 3–4 hours until the aggregates looked visually clean. The dissolved asphalt mixture was centrifuged at approximately 15,000 m/s^2^ for 20 min at 20 °C (± 3 °C). The solvent was removed from the extracted bitumen–toluene solution by rotary evaporator according to EN 12697-3 [54]. The majority of the solvent was removed under the 40 kPa pressure and 90 °C. Bitumen recovery was done in four phases: (1) the majority of toluene was evaporated at a pressure of 40 kPa and a temperature of 90 °C; (2) then, the temperature was increased up to 160 °C with 20 °C increments; (3) the pressure was raised up to 2.0 kPa; and (4) recovered bitumen was kept rotating in a 160 °C oil bath for 1 h. During bitumen recovery, nitrogen gas was used in a rotary evaporator with of 0.5 L/min flow to avoid additional bitumen aging.

### 2.2. Tests and Analysis Methods

#### 2.2.1. Penetration and Softening Point Test Procedures

The penetration (Pen) of neat and polymer-modified bitumen after different aging stages as well as recovered bitumen from pavement was determined in accordance with EN 1426 [55] at 25 °C. The softening point (T_R&B_) was determined in accordance with EN 1427 [56]. In this research, the PI was estimated based on EN 12591 [57] Annex A standard regulation. The results of the mean values were used to estimate the PI and are presented in Table 1.

#### 2.2.2. Bitumen Fractional Groups (Sara) Composition Test Procedure

The bitumen fractional groups (saturates, aromatics, resins, and asphaltenes; SARA) were determined using an IATROSCAN MK6s (NTS Instruments, Xiamen, China) thin-layer chromatograph with flame-ionization detection (TLC-FID), referring to IP 469/01 [58]. The samples were prepared dissolving 1% ± 0.1% (m/V) bitumen in toluene. After cleaning the activated quartz rods (chromarods), 1 µL of sample solution was spotted on each rod with a semi-automatic spotter. The frame with 10 rods was placed in the drying chamber at 80 °C for around 2 min to evaporate the residual toluene. The bitumen fractional groups were resolved placing rod frames into three tanks with solvents: Tank A with n-heptane (100%) to elute saturates; Tank B with toluene (80%) and n-heptane (20%) to elute aromatics; and Tank C with dichloromethane (95%) and methanol (5%) to elute resins. Finally, the quartz rods were scanned in the TLC-FID analyzer IATROSCAN MK6s (NTS Instruments, Xiamen, China). For each quartz rod, the four peak areas were integrated at the lowest point before and after each peak, after which the percent concentrations for saturates, aromatics, resins, and asphaltenes were calculated. 

Colloidal instability (Gaestel) index I_c_ was calculated from SARA fractions, and with aging showed increased content level of asphaltenes [12,24,59]. The I_c_ is usually used to compare the stability of different bitumens. I_c_ shows the dispersing capability of maltenes to asphaltenes [11] according to Equation (3).

#### 2.2.3. Attenuated Total Reflection (ATR) Fourier Transform Infrared (FT-IR) Spectroscopy Procedure

The infrared spectra was determined using a portable Bruker ALPHA Fourier Transform Infrared (FT-IR) Spectroscope with a single reflection diamond attenuated total reflection (ATR). Then, 1 g of bitumen samples was poured on oil-paper to cool down. During measurements, the sample was placed on the internal reflection element and loaded with a fixed load to ensure full contact with the ATR diamond. Each sample was scanned 24 times with resolution of 4 cm^−1^ in the 4000–400 cm^−1^ range, and the averaged spectrum was recorded. 

FT-IR ATR spectroscopy was used to investigate the oxidation levels and polymer in bitumen [60,61,62,63,64,65]. Due to the stimulation with infrared signal waves and the absorption of energy by the molecules, the determined ATR spectra contains information about the composition and the structure of the binder, including the compounds and functional groups of the bitumen components. Most of the studies were focused on several functional groups, i.e., the carbonyls and sulfoxides, and thus on several peaks of the spectra to pursue the aging behaviour of the binder [17]. In addition, this test can be used in order to determine the chemical groups of the aged, modified, or unknown bitumen [24,66]. To determine the oxidation-related changes from FT-IR absorption, the band areas were used. The measured spectra were analysed with BRUKER OPUS software using the following procedure [65,67]:

- Application of atmospheric and water compensation;
- Correction of baseline;
- Smoothing the spectrum line;
- Compute the band areas using valley-to-valley integration.

The peaks value area ($AR_{i}$) was normalized to the total sum of all the band area ($\sum AR_{v}$). The following bitumen functional structures and indexes can be determined from IR spectra [68]:

- Aromatic structures are defined by the aromaticity index $I_{AR}=AR_{1600}/\sum AR_{v}$ ;
- Aliphatic structures are defined by the aliphatic index $I_{AL}=\left(AR_{1460}+AR_{1376}\right)/\sum AR_{v}$, branched $B=AR_{1376}/\left(AR_{1460}+AR_{1376}\right)$, and long chains $L=AR_{724}/\left(AR_{1460}+AR_{1376}\right)$;
- Oxidative aging structures are defined by the carbonyl index $I_{CO}=AR_{1700}/\sum AR_{v}$ and sulfoxide index $I_{SO}=AR_{1030}/\sum AR_{v}$;
- Polymer modification structures are defined by the polybutadiene index $I_{PB}=AR_{968}/\sum AR_{v}$ and polystyrene index $I_{PS}=AR_{700}/\sum AR_{v}$.

The changes in these indices over the aging cycle were analysed as the oxidation rate and bitumen type (neat and modified) determination. 

#### 2.2.4. Rheological Properties Test Procedures

The rheological properties of bitumen were determined using the Anton Paar dynamic shear rheometer (DSR) MRC 302. The samples for DSR testing were prepared by pouring preheated bitumen into a silicon mold, which was kept in constant conditions at 5 °C temperature until testing. The tests were performed with an 8-mm parallel geometry plate and a 2.0-mm gap size. Before tests, the bitumen samples were placed onto a DSR plate and preheated up to 60 °C to assure the adhesion between the sample and plate. The excessive bitumen of the sample was carefully trimmed with a hot spatula. 

The DSR tests performed in this research consisted of two procedures: (1) the strain sweep tests to determine the linear viscoelastic (LVE) range, and (2) the frequency sweep test to determine the dynamic shear modulus (G*) at linear strain.

The LVE range for each bitumen was determined by performing strain sweep tests (1) at 2 °C and (2) 44 °C temperature and 100 rad/s angular frequency. The strain sweep tests were performed starting with 0.01% strains and increasing logarithmically up to 63% strains, where not less than 30 cycles were performed at each strain level. The strain of the LVE range was defined according to the G* decrease with respect to the strain increase by assuming that the limit at G* decreased to 95% of its initial values [69]. 

The strain control frequency sweep (FS) tests were conducted at a temperature range from 44 °C to 2 °C, with 6 °C increments. The FS tests for the shear oscillation were performed at frequencies from 1 to 100 rad/s with logarithmic increment at each temperature and LVE strains. FS tests were performed at 40% lower strains than the determined LVE limit, while the strains at middle temperatures were linearly interpolated at each temperature level. A master curve was obtained from the complex shear modulus $\left|G^{*}\right|$ determined over a range of temperatures and frequencies under the linear range of bitumen behaviour. The reference temperature must be selected to horizontally shift the $\left|G^{*}\right|$ data collected at other temperatures with respect to the loading frequency. Master curves were horizontally shifted at 20 °C using the Gordon–Show method [70] and the generalized sigmoidal function was fitted with an EXCEL Solver by minimizing the root mean square error. Afterwards, the G-R parameter and crossover temperature T_δ = 45°_ were determined from the master curve function. The T_δ = 45°_ was also determined from measured data at a phase angle of 45° and constant frequency of 10 rad/s. 

## 3. Results and Discussion

**Figure 1:**
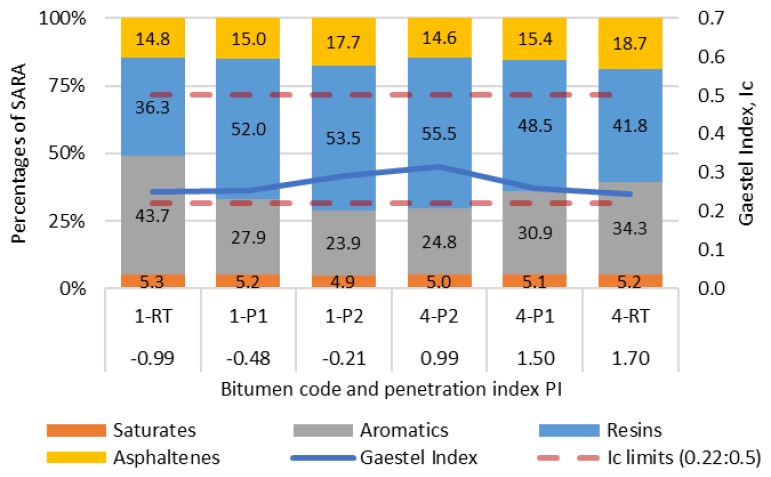
Comparison of SARA fractional groups, penetration index (PI) and instability index (I_c_) due to aging influence.

The results of bitumen fractional composition (SARA) were used to determine the colloidal instability (Gaestel) index I_c_, which defines the overall colloidal system of bitumen. In contrast, the penetration index (PI) is a measure for the bitumen temperature sensitivity, where an increase of PI is associated with a decrease of temperature sensitivity [41]. The mean value of five to 10 rod readings of bitumen fractional groups (SARA) composition are presented in Table 2 and Figure 1. The results showed that the investigated different age bitumen binders are colloidally stable (sol–gel type); to be specific, I_c_ < 0.22–0.5 < I_c_. However, all the recovered binders showed higher instability indexes than the corresponding other bitumen samples. This effect can be not only due to aging but also due to the bitumen recovery procedure. It was determined that the recovered binder did not have the same aging period as the PAV I or PAV II bituminous binders.

Figure 1 shows the comparison of SARA fractional groups, PI and I_c_, due to aging influence. With aging, the content of resins increased since aromatics turns to resins, which later will be generated to asphaltenes. The highest content of asphaltenes was determined for recovered bitumen (natural aging). Asphaltene is the heaviest and most polar bitumen fraction, which has the highest effect on bitumen colloidal stability. The results show that asphaltene content increased with aging for neat bitumen (Figure 1). The opposite effect was determined for SBS-modified bitumen, where the asphaltene content decreased with aging. This phenomenon is assumed to be due to (1) the aging of polymer, which may be attributed as the asphaltenes during the TLC-FID test and which quantity decrease with aging, or (2) after aging, the SBS polymer combines together with asphaltenes in to long-chain and their molecular weight becomes too heavy to solute in toluene and upraise the rod. However, further investigation is needed to determine the effect of the polymer on the SARA results from the TLC-FID test.

The tests show (Table 1 and Figure 1) a penetration decrease and softening point temperature increase with age for neat and polymer-modified (PM) bitumen, but the PI changes conversely increased for neat bitumen and decreased for PM bitumen. The PI increase for neat bitumen is resulted by hardening due to the increased content of asphaltenes and I_c_. However, the PI decrease for PM bitumen needs extensive research to deliver a scientifically proven conclusion, considering that the concept of PI was developed for neat bitumen. The PI gives an approximation of neat bitumen rheological behaviour; however, to indicate more accurate behaviour at extreme temperatures or to predict the behaviour of polymer-modified bitumen the complex shear modulus or viscosity measurements should be performed. 

**Figure 2:**
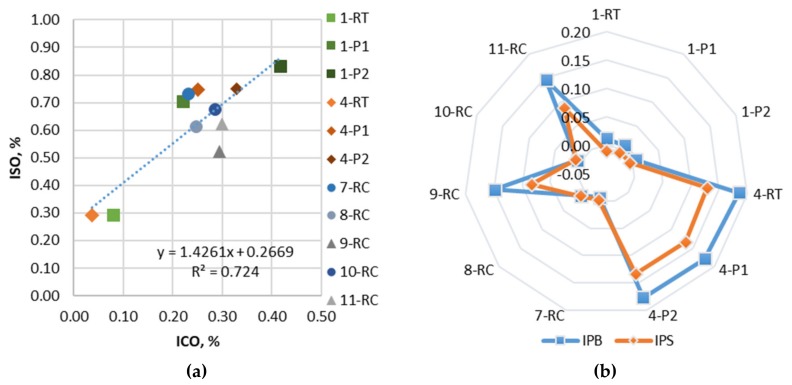
Functional groups representation: **(a**) Relation of carbonyl and sulfoxide indexes; (**b**) The validation of polymer indexes of neat and polymer-modified bitumen under short, long, and double long-term laboratory and natural aging.

The FTIR-ATR test results for the composition of the bitumen functional groups were used to determine the oxidative aging structures (carbonyl I_CO_ and sulfoxide I_SO_ indexes) as well as determine the type of bitumen (neat or modified). A summary of the bitumen functional groups results is presented in Table 3, which shows the mean values of two to four replicates. The differences between the carbonyl and sulfoxide indexes are presented in Figure 2a. The results show that I_CO_ increases all the time with aging, but I_SO_ mainly increases after the first PAV; then, it stabilizes and changes slightly. The I_CO_ increased by 2.8 times after PAV I (1-P1) and 5.2 times after PAV II (1-P2) for neat bitumen, and by 6.8 times after PAV I (4-P1) and 8.9 times after PAV II (4-P2) for modified bitumen. The I_SO_ increased also by 2.4 times after PAV I (1-P1) and 2.8 times after PAV II (1-P2) for neat bitumen, and by 2.6 times after PAV I (4-P1) and 2.6 times after PAV II (4-P2) for modified bitumen. However, polymer-modified bitumen after the same double long-term aging PAV II (44 h) showed a lower content of carbonyl and sulfoxide indexes, which indicates that modified bitumen aging less. The correlation between SO and CO indexes (Figure 2a) showed that unknown naturally aged bitumen had similar values to bitumen after 22 h PAV (P1). Therefore, for further research, in order to evaluate the effect of long-term aging in Lithuania climatic conditions, we can assume 24 h of PAV, which will represent around 15 years of pavement service life. 

The FTIR-ATR results showed that two out of five recovered bitumen (form field-aged pavement) contain polymer structures (Figure 2b). The bitumen 7-RC, 8-RC, and 10-RC are neat bitumen, because the I_PB_ and I_PS_ indices are minor, whereas 9-RC and 11-RC are polymer-modified bitumen, with I_PB_ = 0.148–0.146% and I_PS_ = 0.083–0.087%. However, the polymer content of recovered bitumen is different compared to modified bitumen 4 (PMB 45/80-55). The polybutadiene index was 17.3–18.4% less and the polystyrene index was 38.5–35.6% less compared to recovered and laboratory-aged polymer modified bitumen. The polymer degradation effect due to aging was not determined, since bitumen 4 after the RTFOT (RT), 22 h PAV (P1), and 44 h PAV (P2) had no significant changes in I_PB_ and I_PS_. 

**Figure 3:**
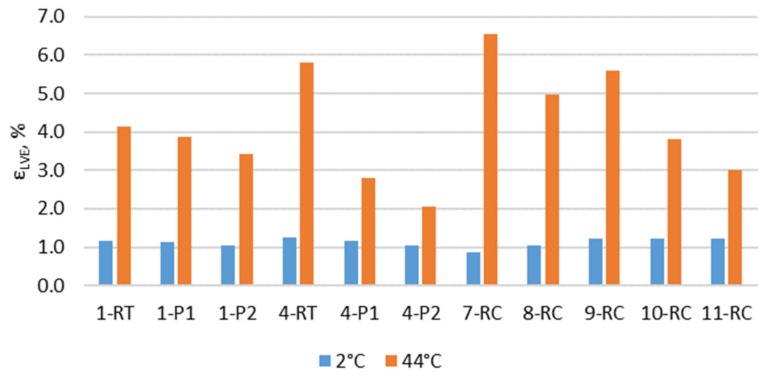
The strain limits of the linear viscoelastic range.

To determine the master curves and to use the time–temperature superposition principles, the LVE region was determined. The strain of LVE range (ε_LVE_) determined at 95% decreased the initial G* value during the strain sweep test (with 8-mm geometry) and is presented in Figure 3. The strain at 2 °C was around 1.13% to all bitumen binders; however, the biggest difference was determined at 44 °C. The test results show that with increasing aging steps the ε_LVE_ decreases; however, this was an expectation for the recovered binder. 

**Figure 4:**
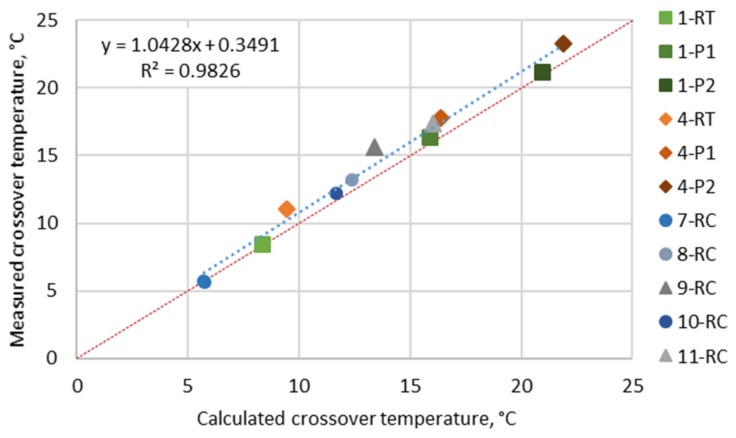
Correlation of measured crossover temperatures by temperature frequency sweep test and calculated from the master curve.

The crossover point (described as temperature or frequency) represents the balance between the storage (G′) and loss (G″) modulus, which means the transition point of colloidal stability; then, the gel–sol turns to sol–gel behaviour. For analysis, the crossover temperature was selected considering the fact that at temperatures above T_δ = 45°_, bitumen behaviour dominates in sol–gel. It is expected that flow stress and rutting damage appear, while below T_δ = 45°_, tension strain and cracking appear [51]. According to some authors [51], the crossover temperature (T_δ = 45°_) shifts with bitumen age as a result of the increased amount of larger molecular weight functional groups. Importantly, T_δ = 45°_ can be determined calculating it from a master curve at a 45 °C phase angle and 10 rad/s, or it can be obtained directly from the temperature sweep experimental results. Next, we consider that the T_δ = 45°_ can be also used to validate the master curve function fitting [51]. The correlation of measured crossover temperatures by a temperature–frequency sweep test and calculations from a master curve is presented in Figure 4. From Figure 4, the T_δ = 45°_ increases with aging can be seen: neat bitumen (1-RT) T_δ = 45°_ increased by 7.9 °C after PAV I (1-P1) and 12.8 °C after PAV II (1-P2), while polymer-modified bitumen (4-RT) increased by 6.7 °C after PAV I (4-P1) and 12.2 °C after PAV II (4-P2). The bitumen 7-RC, 8-RC, and 10 RC show lower T_δ = 45°_ than expected comparing to their life in service, which may be due to a properly performed recovery procedure and the remaining extracted solvent. The correlation between measure and calculated T_δ = 45°_ is very good, but slightly up from the control line.

**Figure 5:**
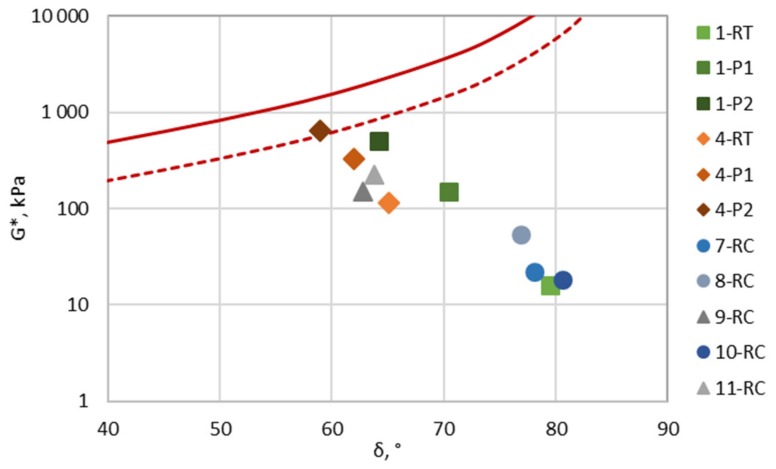
Glover–Rowe (G-R) parameter Black Space plot with damage zone (red line damage zone G-R = 450 kPa; red dotted line warning zone G-R = 180 kPa).

The neat and modified bitumen were analysed approaching the warning and damage limits (zones) to assess the pavement resistance to ravelling and cracking susceptibility at intermediate temperatures, as proposed by Rowe at al. [50]. The Glover–Rowe (G-R) parameter was calculated from the master curves at 15 °C and 0.005 rad/s of tested bitumen, based on Equation (5). The complex shear modulus (G*) and phase angle (*δ*) are the main representatives of G-R, the changes which changes—alongside a comparison of the aging impact—are best analysed in a Black Space plot. The G-R parameter damage zones of the ductility-based failure planes and bitumen G* versus *δ* change due to aging in Black Space are presented in Figure 5. The laboratory aged neat (1) and modified (4) bitumen with increasing aging approach the G-R warning limit (G-R = 180 kPa); however, only modified bitumen after PAV II (4-P2) crosses it. Unfortunately, the rheological properties of recovered bitumen (7-RC, 8-RC, 9-RC, 10-RC and 11-RC) were highly affected by the partial evaporation of toluene, which resulted in an incommensurate low G-R parameter. The representation of the G-R parameter in a Black Space diagram is a promising indicator to evaluate bitumen aging and predict the relative surface damage for bought neat and modified bitumen. However, the additional field validation of the G-R parameter has to be provided, taking in consideration the precise and accurate bitumen recovery procedure. 

## 4. Conclusions

The study presented in this paper analyzed the aging effects of neat (70/100) and SBS polymer-modified (PMB 45/80-55) bitumen, and unknown bitumen recovered from 12–19 years’ pavement in service. The bitumen was tested with a thin-layer chromatography flame ionisation detector, fourier transform infrared spectroscope in attenuated total reflectance mode to determine the effect of aging on the chemical properties, and a penetrometer, softening point tester, and dynamic shear rhoemeter were used to analyze the rheological properties. The results and applied data analysis methods promote the understanding of the effects of aging on the performance of bitumen. The following conclusions summarize the work done in this research:

1. The instability (Gaestel) index I_c_ determined by SARA analysis showed the colloidal stability of all tested bitumen specimens. Laboratory tests confirm the Gastel index increasing with neat bitumen aging; hence, this is not applicable for polymer-modified bitumen specimens, as I_c_ decreased for the PMB binder after aging. The authors state that this is due to polymer chain rupture, which leads to some ruptured parts allocated to resins. It is important to note that polymer chains are mostly ruptured after PAV I (22 h), and there is minor difference in asphaltenes content change after PAV II (44 h).
2. The results of oxidative aging structures, according to the carbonyl I_CO_ and sulfoxide I_SO_ indexes showed that after the first long-term period of aging, the sulfoxide I_SO_ index increases and stabilizes, but the carbonyl I_CO_ index increases with every aging step. The recovered naturally aged bitumen sulfoxide and carbonyl indexes were close to bitumen after 22 h of PAV aging. Thus, for further research to evaluate the effect of long-term aging in northern climatic conditions, researchers can expect to use 24 h of PAV, which will represent around 15 years of pavement service life.
3. The aging influence on the rheological properties of bitumen was investigated through the penetration index (PI) and crossover temperature (T_δ = 45°_), as well as the Glover–Rowe (G-R) parameter determined from master curves, which allowed comparing different types and ages bitumen. The rheological property of recovered bitumen was affected by the toluene solvent residual, so the rheological property results of 7-RC, 8-RC, 9-RC, 10-RC, and 11-RC were neglected. For further investigation, a more accurate bitumen recovery procedure has to be established.
4. Crossover temperature (T_δ = 45°_) represented the transition point of colloidal stability; then, the gel–sol turns to sol–gel behavior. The increase of crossover temperature is related with neat and modified bitumen hardening, because the storage (elastic) modulus G’ became dominant at a higher temperature, which eventually affects the decrease of pavement resistance to thermal and fatigue cracking. The T_δ = 45°_ of neat bitumen 70/100 8.4 °C increased by 7.9 °C (up to 16.4 °C) after PAV I and by 12.8 °C (up to 21.2 °C) after PAV II, and modified bitumen PMB 45/80-55 11.1 °C increased by 6.7 °C (up to 17.8 °C) after PAV I and by 12.2 °C (up to 23.3 °C) after PAV II. So, the modified bitumen PMB 45/80-55 T_δ = 45°_ changed at a 40.5% smaller range compared to neat 70/100 bitumen; this indicates that the neat bitumen sensitivity to thermal effects changes with age faster than modified bitumen.
5. The G-R parameter of complex shear modulus (G*) and phase angle (at 15 °C and 0.005 rad/s) represents the warning zone of non-load-associated cracking (G-R ≤ 180 kPa) and the damage zone of significant cracking (G-R ≤ 450 kPa). The phase angle (*δ*) of neat bitumen 70/100 79.5° after RTFOT decreased by 15.4° (up to 64.2°) after PAV II, while modified bitumen PMB 45/80-55 *δ* 65.2° decreased by 6.2° (up to 58.9°). The complex shear modulus (G*) of 70/100 16.1 kPa increased by 481.2 kPa (up to 499.2 kPa), while the G* of PMB 45/80-55 111.6 kPa increased by 533.2 kPa (up to 644.8 kPa). Test results showed that the changes in the neat bitumen rheological properties due to aging were more significant than those of modified bitumen, however only modified bitumen PMB 45/80-55 after PAV II G* and *δ* values were 10.9% above the G-R warning zone. Therefore, the ravelling and cracking failures after long-term aging occurrence were more likely for PMB 45/80-55 bitumen comparing to 70/100. However, for more specific conclusions, additional research has to be done, investigating more samples and conducting field validation of the G-R parameters.
6. It has to be noted that when using different parameters to evaluate the aging properties of bitumen, quite conflicting conclusions may be obtained because each index only represents a certain aspect of the bitumen properties, which is mainly dominated by the colloidal system of specific bitumen. In order to avoid this, a database of different aged bitumen properties has to be collected.

## Figures and Tables

**Table 1:** Summary of materials and results of penetration (Pen) and softening point (T_R&B_).

Code	Bitumen Penetration Grade	Aging and Sample Preparation Method	Pen at 25 °C, mm^−1^	T_R&B_, °C	Penetration Index (PI)
1-RT	70/100	RTFOT	52.4	50.5	−0.99
1-P1	70/100	PAV I (22 h)	33.3	57.1	−0.48
1-P2	70/100	PAV II (44 h)	26.1	61.1	−0.21
4-RT	PMB 45/80-55	RTFOT	41.1	66.3	1.70
4-P1	PMB 45/80-55	PAV I (22 h)	28.6	70.0	1.50
4-P2	PMB 45/80-55	PAV II (44 h)	23.1	69.5	0.99
7-RC	Unknown	Recovered	54.0	51.8	−0.58
8-RC	Unknown	Recovered	43.2	54.1	−0.58
9-RC	Unknown	Recovered	47.0	61.1	1.09
10-RC	Unknown	Recovered	58.7	51.9	−0.36
11-RC	Unknown	Recovered	37.4	62.0	0.71

**Table 2:** Summary of SARA results. SARA: saturates, aromatics, resins, and asphaltenes.

Code	Bitumen Grade	Aging and Sample Preparation Method	SARA Fractional Groups, %				Gaestel Index I_c_
			Saturates	Aromatics	Resins	Asphaltenes	
1-RT	70/100	RTFOT	5.28	43.66	36.30	14.76	0.251
1-P1	70/100	PAV I (22 h)	5.21	27.86	51.95	14.97	0.253
1-P2	70/100	PAV II (44 h)	4.94	23.87	53.54	17.66	0.292
4-RT	PMB 45/80-55	RTFOT	5.18	34.32	41.76	18.73	0.314
4-P1	PMB 45/80-55	PAV I (22 h)	5.12	30.94	48.54	15.40	0.258
4-P2	PMB 45/80-55	PAV II (44 h)	5.03	24.82	55.55	14.60	0.244
7-RC	Unknown	Recovered	7.68	30.56	41.70	20.05	0.384
8-RC	Unknown	Recovered	6.01	31.52	44.72	17.75	0.312
9-RC	Unknown	Recovered	6.50	31.67	43.14	18.68	0.337
10-RC	Unknown	Recovered	6.79	33.23	42.09	17.88	0.328
11-RC	Unknown	Recovered	6.70	29.55	44.58	19.18	0.349

**Table 3:** Summary of bitumen functional groups results.

Code	Bitumen Grade	Aromatic Structures	Aliphatic Structures			Oxidative Aging Structures		Polymer Structures	
		I_AR_,%	I_AL_,%	B,%	L,%	I_CO_,%	I_SO_,%	I_PB_,%	I_PS_,%
1-RT	70/100	0.88	11.26	0.09	0.34	0.080	0.292	0.012	−0.011
1-P1	70/100	1.09	8.78	0.25	0.80	0.221	0.704	0.009	−0.006
1-P2	70/100	1.16	8.73	0.47	0.94	0.418	0.831	0.007	−0.004
4-RT	PMB 45/80-55	0.98	9.64	0.04	0.33	0.037	0.293	0.186	0.129
4-P1	PMB 45/80-55	1.05	9.18	0.29	0.85	0.251	0.747	0.179	0.135
4-P2	PMB 45/80-55	1.13	8.91	0.37	0.85	0.329	0.749	0.177	0.134
7-RC	Unknown	1.03	9.97	0.27	0.84	0.232	0.732	−0.007	−0.002
8-RC	Unknown	1.08	9.60	0.28	0.70	0.248	0.614	0.009	0.010
9-RC	Unknown	0.32	18.74	0.37	0.67	0.295	0.523	0.148	0.083
10-RC	Unknown	1.03	9.88	0.33	0.77	0.286	0.674	0.007	0.009
11-RC	Unknown	0.67	15.60	0.37	0.76	0.299	0.622	0.146	0.087
